# Peripapillary Retinal Nerve Fiber Layer Profile in Relation to Refractive Error and Axial Length: Results From the Gutenberg Health Study

**DOI:** 10.1167/tvst.9.9.35

**Published:** 2020-08-21

**Authors:** Felix Mathias Wagner, Esther Maria Hoffmann, Stefan Nickels, Achim Fiess, Thomas Münzel, Philipp S. Wild, Manfred E. Beutel, Irene Schmidtmann, Karl J. Lackner, Norbert Pfeiffer, Alexander Karl-Georg Schuster

**Affiliations:** 1 Department of Ophthalmology, University Medical Center of the Johannes Gutenberg-University Mainz, Mainz, Germany; 2 Center for Cardiology – Cardiology I, University Medical Center of the Johannes Gutenberg-University Mainz, Mainz, Germany; 3 German Center for Cardiovascular Research (DZHK), partner site Rhine-Main, Mainz, Germany; 4 Preventive Cardiology and Preventive Medicine, Center for Cardiology, University Medical Center of the Johannes Gutenberg-University Mainz, Mainz, Germany; 5 Center for Thrombosis and Hemostasis (CTH), University Medical Center of the Johannes Gutenberg-University Mainz, Mainz, Germany; 6 Department of Psychosomatic Medicine and Psychotherapy, University Medical Center of the Johannes Gutenberg University Mainz, Mainz, Germany; 7 Institute of Medical Biostatistics, Epidemiology and Informatics (IMBEI), University Medical Center of the Johannes Gutenberg-University Mainz, Mainz, Germany; 8 Institute of Clinical Chemistry and Laboratory Medicine, University Medical Center of the Johannes Gutenberg-University Mainz, Mainz, Germany

**Keywords:** retinal nerve fiber layer, optical coherence tomography, population-based cohort

## Abstract

**Purpose:**

To investigate the retinal nerve fiber layer profile measured by optical coherence tomography and its relation to refractive error and axial length.

**Methods:**

The Gutenberg Health Study is a population-based study in Mainz, Germany. At the five-year follow-up examination, participants underwent optical coherence tomography, objective refraction and biometry. Peripapillary retinal nerve fiber layer (pRNFL) was segmented using proprietary software. The pRNFL profiles were compared between different refraction groups and the angle between the maxima, i.e., the peaks of pRNFL thickness in the upper and lower hemisphere (angle between the maxima of pRNFL thickness [AMR]) was computed. Multivariable linear regression analysis was carried out to determine associations of pRNFL profile (AMR) including age, sex, optic disc size, and axial length in model 1 and spherical equivalent in model 2.

**Results:**

A total of 5387 participants were included. AMR was 145.3° ± 23.4° in right eyes and 151.8° ± 26.7° in left eyes and the pRNFL profile was significant different in the upper hemisphere. The AMR decreased with increasing axial length by −5.86°/mm (95% confidence interval [CI]: [−6.44; −5.29], *P* < 0.001), female sex (−7.61°; 95% CI: [−8.71; −6.51], *P* < 0.001) and increased with higher age (0.08°/year; 95% CI: [0.03; 0.14], *P* = 0.002) and larger optic disc size (2.29°/mm^2^; 95% CI: [1.18; 3.41], *P* < 0.001). In phakic eyes, AMR increased with hyperopic refractive error by 2.60°/diopters (dpt) (95% CI: [2.33; 2.88], *P* < 0.001).

**Conclusions:**

The pRNFL profiles are related to individual ocular and systemic parameters.

**Translational Relevance:**

Biometric parameters should be considered when pRNFL profiles are interpreted in diagnostics, i.e., in glaucoma.

## Introduction

Optical coherence tomography (OCT) has become a routine examination in ophthalmology to assess structural characteristics of the macula and the optic nerve head. Particularly in glaucoma diagnostics, peripapillary retinal nerve fiber layer (pRNFL) thickness is used to detect structural changes being typical for glaucoma,^1^ namely rarefaction of the pRNFL, initially starting in the inferior-temporal (IT) and superior-temporal (ST) area of the optic nerve head.^2^

The pRNFL thickness and its alterations are compared to templates derived from normative databases based on the average of a large sample of subjects^3^ and has been evaluated in several population-based studies,^4^^–^^7^ in addition to smaller clinical studies.^8^^–^^10^ Nevertheless, not only its thickness,^11^ but also its spatial distribution shows a relationship to axial length. In a retrospective case series of 50 eyes, a shift of the pRNFL maxima toward temporal location and a smaller angle between the superior-temporal and inferior-temporal pRNFL maxima was described with increasing axial length.^12^

To date there is no population-based study analyzing the spatial distribution of pRNFL thickness and its distribution in relation to refraction and axial length. This study aims to investigate this relationship and hypothesizes that there is a temporal shift of pRNFL maxima with increasing myopic refractive error and increasing axial length. In addition, the association to sex, age, and optic disc size is investigated.

## Methods

The Gutenberg Health Study (GHS) is a prospective, population-based, observational, single-center cohort study that is being carried out in the Rhine-Main region of Germany (Rhineland-Palatinate). The sample was drawn randomly from local governmental registry offices. The sample was equally stratified for sex, residence (urban or rural), and for each decade of age between the age of 35 to 74 years at study inclusion. The study protocol and study documents were approved by the local ethics committee of the Medical Chamber of Rhineland-Palatinate, Germany (reference no. 837.020.07; original vote 22.3.2007). According to the tenets of the Declaration of Helsinki, written informed consent was obtained from all participants before entering the study. The baseline examination was carried out between 2007 and 2012 and included 15,010 subjects with a consecutive five-year follow-up examination between 2012 and 2017.

At the five-year follow-up, all participants underwent a standardized ophthalmologic examination, including distant-corrected visual acuity and measurement of objective refraction (Humphrey Automated refractor/Keratometer 599), intraocular pressure (with a noncontact tonometer, NT 2000; Nidek Co., Tokyo, Japan), biometry (Lenstar LS900; Haag-Streit, Bern, Switzerland), and nonmydriatic fundus photography. Imaging of the macula and the optic nerve head was carried out with spectral domain (SD)-OCT (Spectralis-OCT, Heidelberg Engineering, Heidelberg, Germany). The spherical equivalent was calculated by adding the spherical correction value to half the cylinder value. Phakic eyes were defined as those eyes with a measurement of ≥2mm lens thickness in optical biometry. More details of the study design have been described by Höhn et al.^13^ Horizontal and vertical optic disc diameter was measured on optic nerve head images, and optic disc size was computed as an ellipse including magnification correction according to Littmann et al.^14^

### Study Sample

This is a cross-sectional analysis of the five-year follow-up visit (2012 –2017). A total of 12,423 subjects of the original cohort presented for the five-year follow-up examination (82.8% of the original cohort, n = 15,010). Of them, only 7568 underwent OCT-imaging due to technical reasons, and 6139 (81%) had sufficient OCT-imaging of the peripapillary RNFL in at least one eye.

In addition, optical biometry and objective refractive measurement were inclusion criteria. For evaluation of the association with refractive error, pseudophakic and aphakic eyes were excluded. The characteristics of the study sample have been described in detail elsewhere.^13^

### Optical Coherence Tomography

The pRNFL was imaged with a SD-OCT (Spectralis-OCT; Heidelberg Engineering, Heidelberg, Germany) with a diameter of 12° (corresponding to 3.47 mm in the standard eye)^15^ and a standard corneal curvature of 7.7 mm. Automated segmentation of the pRNFL was computed with a Heidelberg Eye Explorer (version 1.9.14.0; HEYEX, Heidelberg, Germany), and its thickness was determined. This was automatically carried out at 768 positions of the peripapillary circle (this corresponds to 0.47° per measurement position). All segmented peripapillary OCT scans were manually reviewed by a board-certified ophthalmologist (AF) for quality control. Decentered scans and those with segmentation errors were excluded, i.e., centering more thanone-quarter optic disc-diameter apart from center. Correctly segmented OCT scans on top of an area of peripapillary atrophy or tilted disc were not excluded. A detailed protocol for OCT scan evaluation has been described by Hoffmann et al.^4^

We adjusted the pRNFL measures for ocular magnification incorporating corneal curvature and spherical equivalent.^16^ These parameters are also used in the HEYEX software to adjust for ocular magnification (personal communication with Heidelberg Engineering).

**Figure 1.**
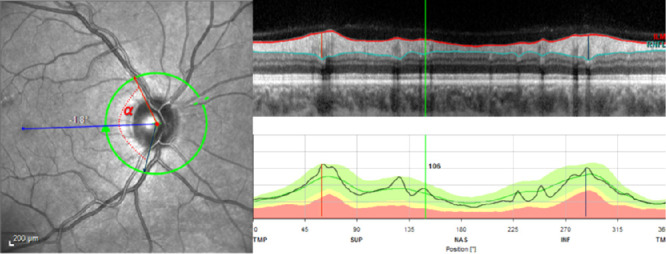
Example of the angle between the pRNFL thickness maxima (peak) of the upper and lower hemisphere (AMR). The pRNFL maxima of the upper hemisphere is illustrated by a *red line* and of the lower hemisphere by a *blue line*. The angle AMR is shown as *α*.

First, pRNFL profiles around the optic disc were computed for right and left eyes for different refractive error (five groups of 3 dpt spherical equivalent steps) and different axial lengths (four groups of 2 mm axial length steps) and graphically compared. Then, we calculated the maxima (peak) of pRNFL thickness in the superior and in the inferior hemisphere of the peripapillary scan circle. The temporal angle (in degrees) between the positions of these two maxima was defined as the angle between the maxima of pRNFL thickness (AMR) (Fig. 1). A sensitivity analysis was performed excluding subjects with self-reported glaucoma (Supplementary Table S2). In addition, we computed the median position of the upper 10% of the measures in each hemisphere as sensitivity analysis and calculated their corresponding temporal angle (Supplementary Fig. S1). This was conducted to consider a split pRNFL distribution, i.e., having two peaks instead of one at the superotemporal or inferotemporal areas. We performed a centering analysis on 500 persons (1000 eyes) and derived a subset of 87 patients (174 eyes) with perfect centration (≤2 pixels of decentration on the infrared images, this corresponds to 50µm) (Supplementary Fig. S2).

### Statistical Analysis

For statistical analysis, absolute and relative frequencies were computed for categorical variables. Median, interquartile range, minimum, and maximum were calculated for all continuous variables. For variables found to be within normal distribution, mean and standard deviation were computed.

Association analysis of AMR was carried out using univariate and multivariable linear regression with generalized estimating equations to consider two eyes of one study participant. As independent variables, age, sex, axial length, corneal curvature, and optic disc size were included in model 1. In model 2, the associations of AMR with age, sex, spherical equivalent and optic disc size was computed in phakic eyes. The pRNFL profiles (each of the 768 measurement points) were compared between right and left eyes using t-test and applying Bonferroni correction (thus a *P* value < 0.00001 was considered as statistically significant). Data were processed by statistical analysis software (R, version 3.5.2.; http://www.R-project.org/, provide in the public domain by R Core Team, Vienna, Austria.). This is an explorative study; thus *P* values should be interpreted as a continuous measure of the compatibility between the data and the entire model used to compute it.^17^

## Results

A total of 5387 subjects (4748 right eyes, 4479 left eyes; 2756 men, 2631 women) were included in this cross-sectional analysis. At the five-year follow-up, 12,423 of the initial 15,010 subjects participated. Of these, 7568 participants had peripapillary OCT scans. Finally, 5387 participants were included in the analysis passing quality assurance of OCT scans and having data on spherical equivalent and corneal curvature. The characteristics of the sample are given in Table 1. Item nonresponder analysis revealed that included subjects were 2.5 years younger compared with those without pRNFL measurement at five-year follow-up examination and were less likely to have diabetes, arterial hypertension, and lower body mass index. They were less likely to be pseudophakic and to have glaucoma (Supplemental Table S1).

**Table 1.** Characteristics of the Analysis Sample Having OCT Imaging of the Peripapillary RNFL Thickness

Characteristic	Overall (n = 5387)	Male (n = 2756)	Female (n = 2631)
Age, mean (SD)	57.94 (10.70)	58.43 (10.78)	57.43 (10.60)
Sex: female (%)	2631 (48.8)	0 (0.0)	2631 (100.0)
SES (median [IQR])	13.00 [10.00, 17.00]	14.00 [11.00, 18.00]	12.00 [10.00, 16.00]
Hypertension (%)	2672 (49.6)	1518 (55.1)	1154 (43.9)
Diabetes mellitus (%)	490 (9.1)	308 (11.2)	182 (6.9)
Body mass index, mean (SD)	27.26 (4.89)	27.85 (4.29)	26.64 (5.39)
Ophthalmologic characteristics			
logMAR right eye (median [IQR])	0.10 [0.00, 0.20]	0.10 [0.00, 0.10]	0.10 [0.00, 0.20]
logMAR left eye (median [IQR])	0.10 [0.00, 0.20]	0.10 [0.00, 0.10]	0.10 [0.00, 0.20]
IOP right eye in mm Hg,(mean (SD)	14.71 (2.88)	14.81 (2.98)	14.61 (2.77)
IOP left eye in mm Hg, mean (SD)	14.81 (2.90)	14.93 (2.99)	14.69 (2.81)
SE right eye in dpt, mean (SD)	‒0.38 (2.18)	‒0.35 (2.12)	‒0.40 (2.23)
SE left eye in dpt, mean (SD)	‒0.38 (2.21)	‒0.35 (2.19)	‒0.41 (2.23)
Axial length right eye in mm, mean (SD)	23.71 (1.15)	23.97 (1.12)	23.44 (1.11)
Axial length left eye in mm, mean (SD)	23.69 (1.16)	23.94 (1.15)	23.42 (1.11)
Corneal radius right eye in mm, mean (SD)	7.77 (0.27)	7.83 (0.27)	7.71 (0.27)
Corneal radius left eye in mm, mean (SD)	7.76 (0.27)	7.82 (0.27)	7.71 (0.26)
Pseudophakia right eye (%)	333 (6.2)	168 (6.1)	165 (6.3)
Pseudophakia left eye (%)	348 (6.5)	174 (6.4)	174 (6.6)
Self-reported glaucoma (%)	174 (3.2)	81 (2.9)	93 (3.5)

Data from the Gutenberg Health Study (2012–2017). SES, socioeconomic status; IQR, interquartile range; SE, spherical equivalent.

**Figure 2.**
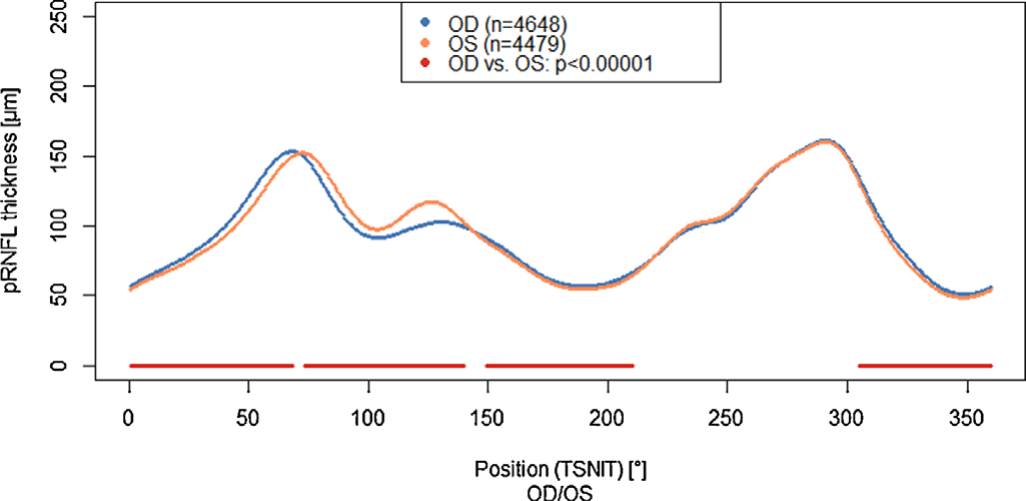
The pRNFL profile and its relation to eye side. Data from the population-based Gutenberg Health Study (2012–2017). The dark red marked positions indicate statistically significant differences (*P* < 10^−5^).

The angle between the maxima of pRNFL thickness in the upper and lower hemisphere (AMR) was 145.3° ± 23.4° in right eyes and 151.8° ± 26.7 ° in left eyes. This difference is also visible in the different pRNFL profiles of right and left eyes (Fig. 2) and in the subgroup of exactly centered pRNFL scans (Supplementary Fig. S2). The pRNFL profiles are statistically significant divergent mostly in the upper hemisphere (Fig. 2).

**Figure 3.**
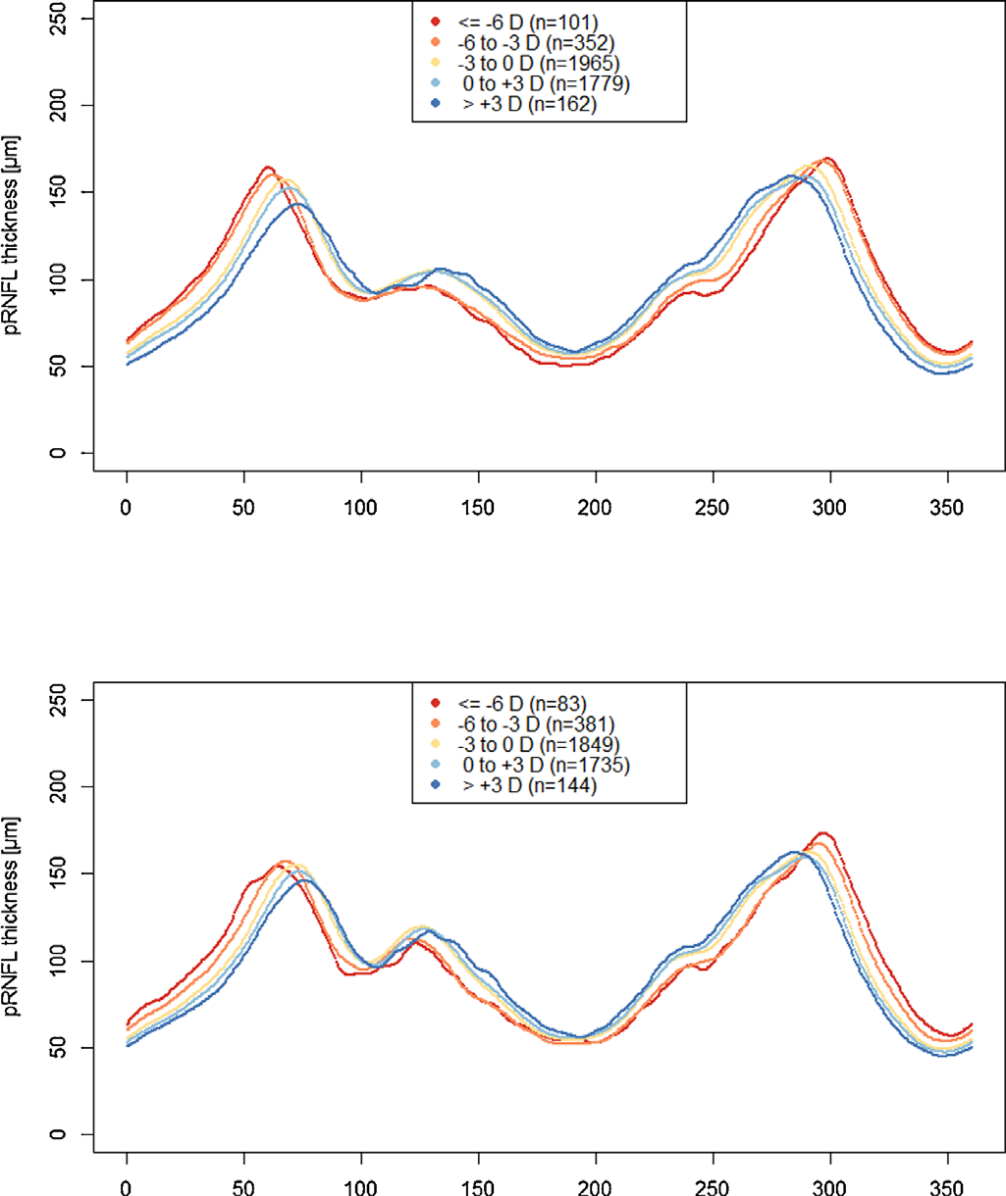
The pRNFL profile and its relation on axial length. Data from the population-based Gutenberg Health Study (2012–2017). (**A**) Right eyes. (**B**) Left eyes.

**Figure 4.**
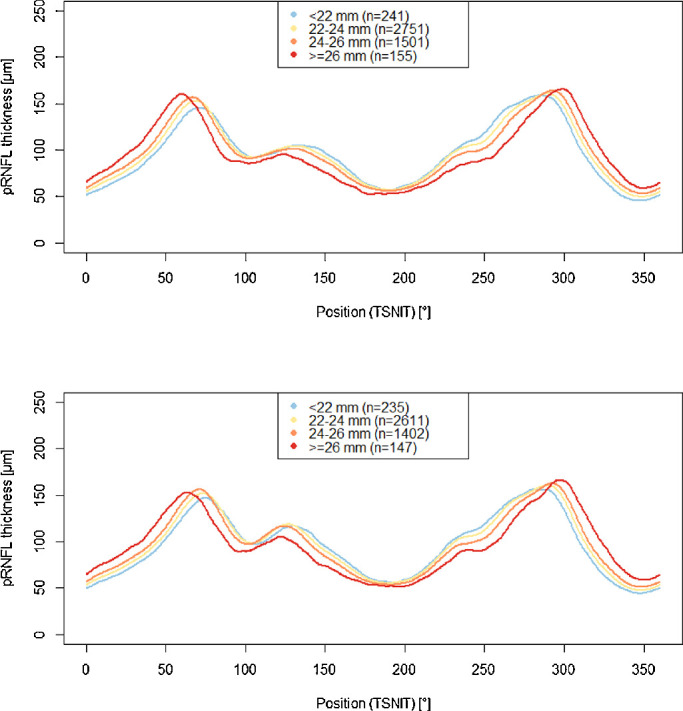
pRNFL profile and its relation to spherical equivalent. Data from the population-based Gutenberg Health Study (2012–2017) including phakic eyes. (**A**) Right eyes. (**B**) Left eyes.

We visually compared the pRNFL profile in relation to axial length for all eyes (Fig. 3): with increasing axial length the angle between the pRNFL thickness maxima in the upper and lower hemisphere decreased. The relation of pRNFL profile and spherical equivalent for phakic eyes are presented in Figure 4: with increasing myopic refractive error the pRNFL thickness maxima are shifted temporally. In Figure 3, the pRNFL thickness is decreased over its entire profile with increasing axial length, in Figure 4 the same is observable for increasing myopic refractive error.

Univariate analysis revealed a statistically significant association between smaller AMR and longer axial length -4.84°/mm (95% CI: [−5.35; −4.34], *P* < 0.001). Furthermore, women had a lower AMR −5.04° (95% CI: [−6.13; −3.96], *P* < 0.001), older age and larger optic disc size were associated with larger AMR, 0.16°/year (95% CI: [0.11; 0.21], *P* < 0.001) and 1.98°/mm^2^ optic disc area (95% CI: [0.85; 3.10], *P* < 0.001), respectively (Table 2). In phakic eyes spherical equivalent was positively associated with AMR 2.59°/dpt (95% CI: [2.33; 2.85], *P* < 0.001) (Table 3).

**Table 2.** Model 1—Association Analysis of pRNFL Profile (Angle Between the Maximal pRNFL Thickness in the Upper and Lower Hemisphere) and Ocular and Systemic Parameters Including Axial Length

	Univariate			Multivariable		
Parameter (n = 8361)	B	95% CI	*P* Value	B	95% CI	*P* Value
Sex (female)	−5.04	−6.13; −3.96	<0.001	−7.61	−8.71; −6.51	<0.001
Age (years)	0.16	0.11; 0.21	<0.001	0.08	0.03; 0.14	0.002
Axial length (mm)	−4.84	−5.35; −4.34	<0.001	−5.86	−6.44; −5.29	<0.001
Corneal curvature (mm)	−4.16	−6.17; −2.15	<0.001	2.05	−0.28; 4.38	0.08
Optic disc size (mm^2^)	1.98	0.85; 3.10	<0.001	2.29	1.18; 3.41	<0.001

Data from the Gutenberg Health Study (2012–2017). Linear regression analysis with generalized estimating equations.

**Table 3.** Model 2—Association Analysis of pRNFL Profile (Angle Between the Maximal pRNFL Thickness in the Upper and Lower Hemisphere) and Ocular and Systemic Parameters in Phakic Eyes

	Univariate			Multivariable		
Parameter (n= 7767)	B	95% CI	*P* Value	B	95% CI	*P* Value
Sex (female)	−5.14	−6.26; −4.02	<0.001	−4.87	−5.97; −3.76	<0.001
Age (years)	0.18	0.13; 0.24	<0.001	0.003	−0.05; 0.06	0.92
Spherical equivalent (dpt)	2.59	2.33; 2.85	<0.001	2.60	2.33; 2.88	<0.001
Optic disc size (mm^2^)	1.66	0.49; 2.82	0.014	1.96	0.83; 3.09	<0.001

Data from the Gutenberg Health Study (2012–2017).

Multivariable analysis showed decreasing AMR with increasing axial length −5.86°/mm (95% CI: [−6.44; −5.29], *P* < 0.001). Women had a lower AMR (−7.61°/mm; 95% CI: [−8.71; −6.51], *P* < 0.001). Older age (0.08°/year; 95% CI: [0.03; 0.14], *P* = 0.002) and larger optic disc size (2.29°/mm^2^; 95% CI: [1.18; 3.41], *P* < 0.001) were associated with a larger AMR, whereas corneal curvature was not associated (Table 2). In phakic eyes, AMR was positively associated with hyperopic refractive error (2.60°/dpt; 95% CI: [2.33; 2.88], *P* < 0.001) in multivariable analysis with adjustment for age, sex, and optic disc size (Table 3).

The sensitivity analysis showed similar associations when excluding subjects with self-reported glaucoma (Supplementary Table S2). Similar associations were observed in the sensitivity analysis analyzing the AMR between the upper 10% of the measures to incorporate, i.e., a split pRNFL distribution (Supplementary Table S3).

## Discussion

Our study is the first population-based study investigating the pRNFL profile and not only sectorial or global pRNFL thickness measures and its associations with ocular and demographic parameters. We demonstrated that pRNFL profile is influenced by refraction of the eye and its axial length. Therefore normative values and clinical evaluation of the pRNFL profile should incorporate the refractive status of the eye or its axial length. With increasing myopia and longer axial length, the superior-temporal and inferior-temporal located pRNFL maxima are shifted temporally.

Previously published studies show associations of pRNFL thickness and axial length or refractive error. Leung et al.^18^ demonstrated that high myopia is associated with a thinner pRNFL. Schuster et al.^12^ described a temporal shift of pRNFL thickness with increasing myopia. An increased axial length leads to a larger fovea to optic nerve head distance, resulting in a temporal shift of the inferior-temporal and superior-temporal maxima measurements.^19^^,^^20^ Consistent with these publications, we found a temporal shift of pRNFL thickness maxima and, accordingly, a decrease of the AMR (angle between the maxima of pRNFL thickness in the upper and lower hemisphere) with increasing myopia.

Our study demonstrates a different pRNFL profile between male and female. We found that female sex relates to a smaller AMR. Older age and larger optic disc size were associated with an increased AMR in our population. Our study confirms findings of Li and Rauscher et al., who previously reported different patterns of pRNFL between male and female subjects. They reported a temporal shift of pRNFL maxima in females that can be translated into an AMR reduction of −4.3° for female sex,^21^ which is very similar to our univariate result of −5.04° (95% CI: [−6.13; −3.96]) for female sex.

Interestingly, we found a difference between the pRNFL profiles of left and right eyes: the AMR was more than 6° higher in right eyes. Sensitivity analyses confirmed this finding. To our knowledge, this is the first time that an intereye difference of pRNFL profile is reported in a population-based study. A possible explanation for this difference could be an asymmetry of retinal vasculature. Leung et al.^22^ reported a slightly but consistently higher mean arteriolar diameter of 2% in the right eye compared with the left eye, but other studies investigating retinal vascular biomarkers such as central retinal arteriolar equivalent, central retinal venular equivalent or arteriovenous ratio could not confirm this finding.^23^^,^^24^ MacGillivray et al.^25^ studied vessel tortuosity and branching geometry in the context of intereye asymmetry and showed only low correlation between right and left eye for these vascular distribution parameters, a review by Cameron et al.^26^ concluded that “these findings do not provide support for the assumption of bilateral equivalence of retinal vascular branching and tortuosity measurements.” Jee et al. reported that the superior retinal vessels in the right eye were located more temporally than in the left eye.^27^ This might be caused i.e. by different branching pattern. The RNFL bundles are located around the large retinal vessels in the upper and lower hemisphere,^28^ especially around the superotemporal and inferior-temporal vessel arcade. When the superior retinal vessels of the right eye are located more temporally, this might lead to a shift of the pRNFL maxima in the upper hemisphere as well. This corresponds to our findings that the AMR of pRNFL is smaller in right eyes than in left eyes and a difference in pRNFL profile is seen in the upper hemisphere with a temporal shift in right eyes compared to left eyes. In addition, not only pRNFL profile, but also its thickness might vary between right and left eyes. Incorporation of retinal blood vessels did lead to lower interindividual variability of pRNFL measures^29^; nevertheless it is not yet incorporated into clinical diagnostics. One cross-sectional study found significant interocular differences of the pRNFL thickness between right and left eye.^30^ These findings of a systematically thicker RNFL in the right eye (particularly temporally) were confirmed by two other OCT-based studies^31^^,^^32^ and one using laser polarimeter.^33^ Consequently, normative values for pRNFL profile should be computed separately for right and left eyes.

Our study has several limitations. First, the majority of our study population originates from Rhineland-Palatinate in south-west Germany and is, hence, a Caucasian population. Therefore the applicability to other ethnicities is limited. Second, there was a high item nonresponder rate. However, item nonresponder analysis revealed that included subjects were compared to the total cohort population at five-year follow-up examination except that they were about 2.5 years younger. Further decentration of peripapillary OCT scans could have affected pRNFL measurement; therefore we performed a sensitivity analysis with perfectly centered OCT scans. All OCT scans were performed with standard setting for corneal curvature of 7.7 mm, as described in other population-based cohort studies, e.g., by Zhao et al. in the Beijing Eye Study.^34^ Furthermore, we included corneal curvature as an adjustment variable but cannot rule out ocular magnification artifacts because the standard RNFL-measurement circle had a diameter of 12°. Further studies should investigate whether a refraction-adjusted circle diameter may explain parts of our findings. The position of the retinal vessels relative to the optic disc was not determined; therefore no statement regarding the difference of vessel profile between right and left eyes could be made. This specific limitation could be solved in the future with the application of OCT angiography. Nonetheless, our study is the first to evaluate associated factors not only for sectorial pRNFL thickness, but for the pRNFL profile in a large population-based sample.

In conclusion, several factors affecting pRNFL profile were identified. First, right and left eyes show a different pRNFL profile. Axial length, refraction, age, sex and optical disc size further affect the pRNFL profile. Our findings indicate that pRNFL profiles are related to individual ocular and systemic parameters. Thus individualized normative pRNFL profiles may lead to improved clinical interpretation of pRNFL, i.e., in glaucoma diagnostics.

## Supplementary Material

Supplement 1

Supplement 2
