# Supplementary material for: Peripapillary Retinal Nerve Fiber Layer Profile in Relation to Refractive Error and Axial Length: Results From the Gutenberg Health Study
Source: Transl Vis Sci Technol. 2020 Aug 21;9(9):35. doi: 10.1167/tvst.9.9.35 (PMC7445357; doi:10.1167/tvst.9.9.35)
Supplement: Supplement 1 [file tvst-9-9-35_s001.pdf]

Figure S1: Example of the angle between the pRNFL thickness maxima (median of the upper 10% measurements) illustrated by a yellow box of the upper and lower hemisphere (AMR). The maxima position of the upper hemisphere is illustrated by a red line and of the lower hemisphere by blue line. The angle AMR is shown as α.

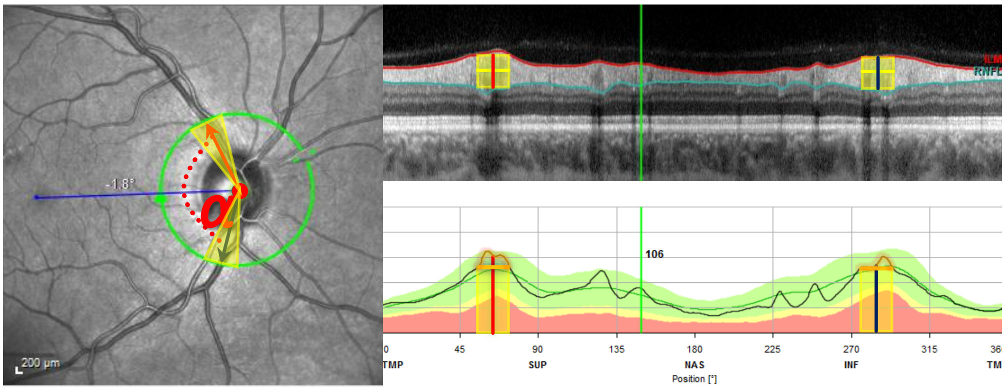

Figure S2: pRNFL profile and its relation to eye side in exactly centered eyes (n(participants) = 87, n(eyes) = 174; left graph) of a random sample of the Gutenberg Health Study (n(participants) = 500 n(eyes) = 1000, right graph). Data from the population-based Gutenberg Health Study (2012-2017).

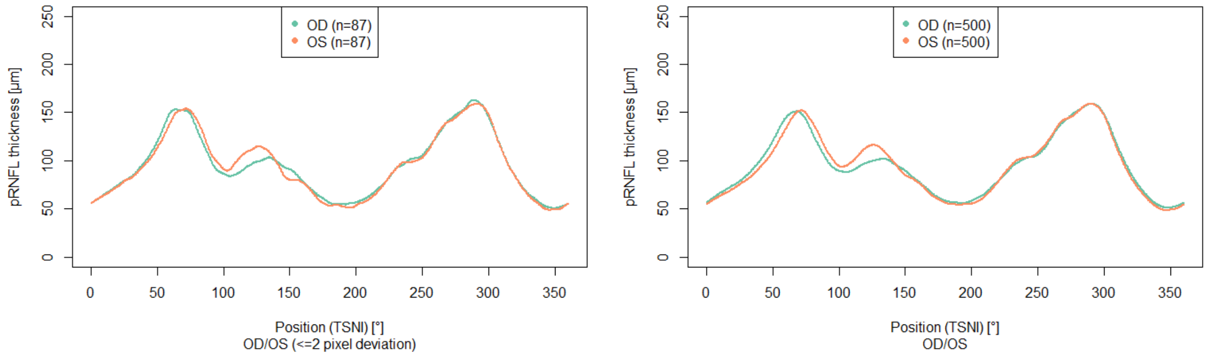

Figure S3: Flow chart of the inclusion of GHS study participants.

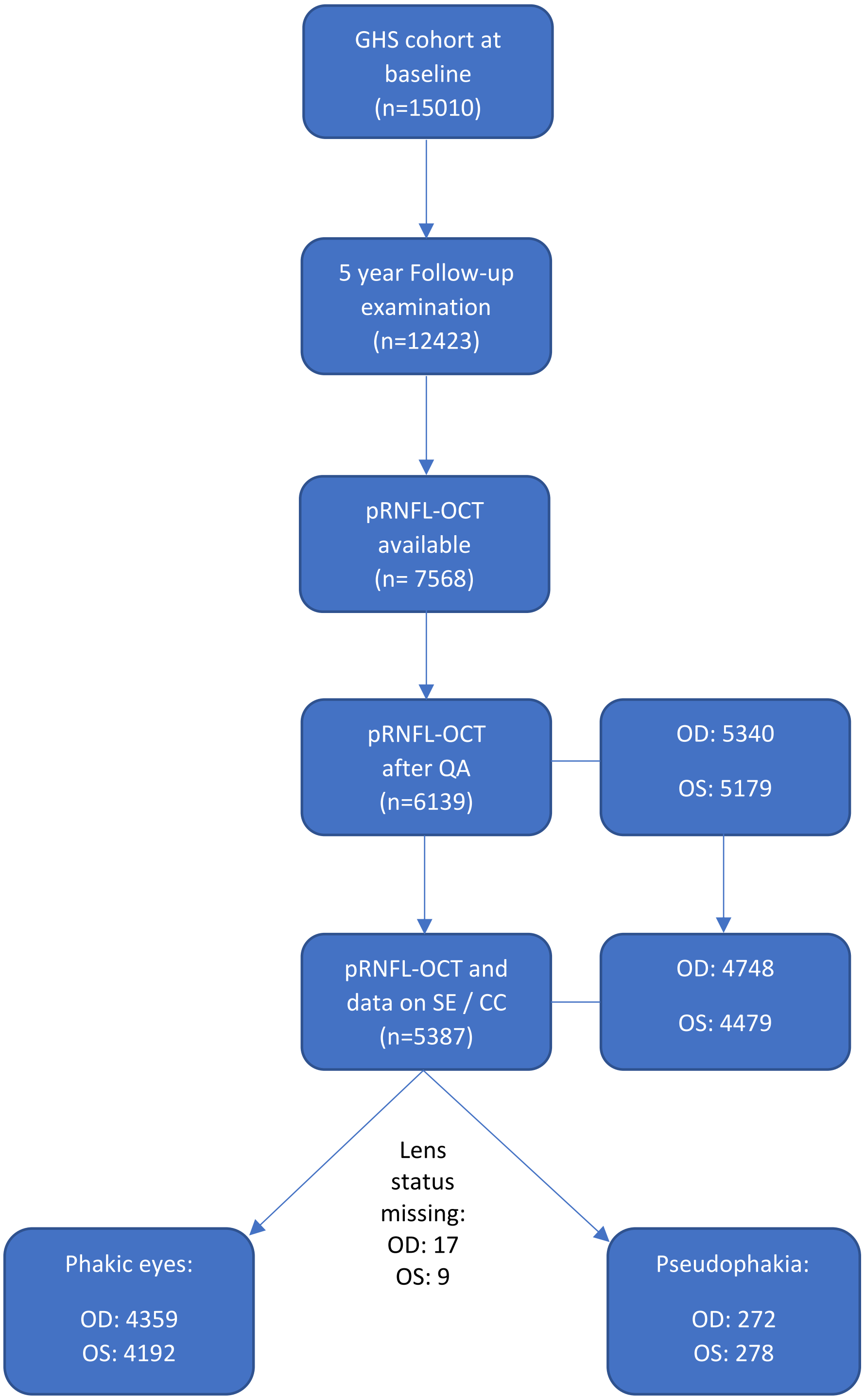

GHS = Gutenberg Health Study ; QA = Quality assessment ;
SE = Spherical equivalent ; CC = corneal curvature
