# Supplementary material for: Peripapillary Retinal Nerve Fiber Layer Profile in Relation to Refractive Error and Axial Length: Results From the Gutenberg Health Study
Source: Transl Vis Sci Technol. 2020 Aug 21;9(9):35. doi: 10.1167/tvst.9.9.35 (PMC7445357; doi:10.1167/tvst.9.9.35)
Supplement: Supplement 2 [file tvst-9-9-35_s002.pdf]

Supplemental Table S1: Item-non-responder analysis with respect to OCT imaging of the peripapillary RNFL thickness. Data from the Gutenberg Health Study (2012-2017)

Characteristic	Item-Responder (n=6139)	Item-Nonresponder (n=6284)	p-value
Age (mean (sd))	58.21 (10.69)	60.74 (10.85)	<0.001
Sex: female (%)	2993(48.8)	3072 (48.9)	0.99
SES (median [IQR])	13.00 [10.00, 17.00]	12.00 [9.00, 16.00]	<0.001
Hypertension (%)	3126 (51.0)	3560 (56.7)	<0.001
Diabetes melitus (%)	573 (9.3)	740 (11.8)	<0.001
Body mass index (mean (sd))	27.34 (4.90)	27.70 (5.13)	<0.001
Ophthalmological characteristcs			
logMAR right eye (median [IQR])	0.10 [0.00, 0.20]	0.10 [0.00, 0.20]	<0.001
logMAR left eye (median [IQR])	0.10 [0.00, 0.20]	0.10 [0.00, 0.20]	<0.001
IOP right eye in mmHg (mean (sd))	14.70 (2.89)	14.82 (3.05)	0.16
IOP left eye in mmHg (mean (sd))	14.79 (2.91)	14.91 (3.13)	0.32
SE right eye in dpt (mean (sd))	-0.36 (2.22)	-0.48 (2.77)	0.14
SE left eye in dpt (mean (sd))	-0.36 (2.23)	-0.47 (2.80)	0.14
Axial length right eye in mm (mean (sd))	23.72 (1.15)	23.79 (1.38)	0.01
Axial length left eye in mm (mean (sd))	23.69 (1.16)	23.74 (1.40)	0.05
Corneal radius right eye in mm (mean (sd))	7.77 (0.27)	7.76 (0.28)	0.05
Corneal radius left eye in mm (mean (sd))	7.76 (0.27)	7.75 (0.28)	0.02
Pseudophakia right eye (%)	394 (6.5)	517 (10.1)	<0.001
Pseudophakia left eye (%)	402 (6.6)	526 (10.3)	<0.001
Self-reported glaucoma (%)	199 (3.2)	296 (4.7)	<0.001

sd = standard deviation
SES = socioeconomic status
IQR = Interquartile range
SE= spherical equivalent

Supplemental Table S2

Association analysis of pRNFL profile (angle between the maximal pRNFL thickness in the upper and lower hemisphere) and ocular and systemic parameters including a) axial length and b) spherical equivalent. Data from the Gutenberg Health Study (2012-2017); eyes with self-reported glaucoma were excluded.

a) All eyes

**n= 8087**	**univariate**			**multivariable**		
Parameter	B	95% CI	P-value	B	95% CI	P-value
Sex (female)	-5.09	-6.20 ; -3.99	<0.001	-7.64	-8.76 ; -6.52	<0.001
Age (years)	0.16	0.11 ; 0.22	<0.001	0.08	0.03 ; 0.14	0.002
Axial length (mm)	-4.85	-5.35 ; -4.34	<0.001	-5.87	-6.45 ; -5.29	<0.001
Corneal curvature (mm)	-4.23	-6.28 ; -2.19	<0.001	1.87	-0.51 ; 4.52	0.12
Optic disc size (mm^2)	2.22	1.08 ; 3.36	<0.001	2.48	1.36 ; 3.61	<0.001

b) Phakic eyes

**n= 7552**	**univariate**			**multivariable**		
Parameter	B	95% CI	P-value	B	95% CI	P-value
Sex (female)	-5.12	-6.34 ; -4.06	<0.001	-4.91	-6.02 ; -3.79	<0.001
Age (years)	0.19	0.13 ; 0.24	<0.001	0.003	-0.06 ; 0.06	0.93
Spherical equivalent (dpt)	2.59	2.33 ; 2.85	<0.001	2.60	2.33 ; 2.88	<0.001
Optic disc size (mm^2)	1.88	0.70 ; 3.06	0.002	2.06	0.91 ; 3.21	<0.001

Supplemental Table S3

Association analysis of pRNFL profile (angle between the median position of the upper 10-percent of the pRNFL thickness measures in the upper and lower hemisphere) and ocular and systemic parameters including a) axial length and b) spherical equivalent. Data from the Gutenberg Health Study (2012-2017);

a) All eyes

**n= 8361**	**univariate**			**multivariable**		
Parameter	B	95% CI	P-value	B	95% CI	P-value
Sex (female)	-4.80	-5.76 ; -3.83	<0.001	-7.43	-8.40 ; -6.47	<0.001
Age (years)	0.14	0.10 ; 0.19	<0.001	0.07	0.02 ; 0.11	0.003
Axial length (mm)	-4.90	-5.36 ; -4.45	<0.001	-5.87	-6.38 ; -5.35	<0.001
Corneal curvature (mm)	-4.74	-6.55 ; -2.93	<0.001	1.40	-0.70 ; 3.50	0.19
Optic disc size (mm^2)	2.07	1.05 ; 3.08	<0.001	2.48	1.49 ; 3.47	<0.001

Linear regression analysis with generalized estimating equations.

b) Phakic eyes

**n= 8716**	**univariate**			**multivariable**		
Parameter	B	95% CI	P-value	B	95% CI	P-value
Sex (female)	-4.79	-5.79 ; -3.80	<0.001	-4.52	-5.49 ; -3.55	<0.001
Age (years)	0.17	0.12 ; 0.22	<0.001	-0.01	-0.06 ; 0.04	0.7
Spherical equivalent (dpt)	2.57	2.34 ; 2.81	<0.001	2.61	2.36 ; 2.85	<0.001
Optic disc size (mm^2)	1.79	0.73 ; 2.85	<0.001	2.11	1.09 ; 3.13	<0.001
